# *Annurca* Apple By-Products at Different Ripening Stages Inhibit AGE Formation and Protect Against AGE-Induced Cytotoxicity Through Antioxidant Activity

**DOI:** 10.3390/antiox15020200

**Published:** 2026-02-03

**Authors:** Maria Liccardo, Pasquale Perrone, Shana Perrella, Ivana Sirangelo, Stefania D’Angelo, Clara Iannuzzi

**Affiliations:** 1 Department of Precision Medicine, Università degli Studi della Campania “Luigi Vanvitelli”, Via L. De Crecchio 7, 80138 Naples, Italy; maria.liccardo@unicampania.it (M.L.); shana.perrella@unicampania.it (S.P.); ivana.sirangelo@unicampania.it (I.S.); clara.iannuzzi@unicampania.it (C.I.); 2 Department of Medical, Human Movement, and Well-Being Sciences (DiSMMeB), Parthenope University of Naples, 80133 Naples, Italy; pasquale.perrone@collaboratore.uniparthenope.it

**Keywords:** *Annurca* apple by-products, AGEs endothelial dysfunction, oxidative stress, antioxidant activity

## Abstract

*Annurca apple* extract is gaining growing attention for its beneficial properties, particularly its outstanding antioxidant activity. Using a combination of biophysical, cell, and molecular biology techniques, this study investigates the sustainable valorization of *Annurca* apple by-products at different ripening stages and their role in the formation of advanced glycation end-products (AGEs), as well as in protection against AGE-related cytotoxicity. AGEs are a class of compounds formed by non-enzymatic reactions between reducing sugars and proteins, lipids, or nucleic acids. They can be produced endogenously or ingested through dietary sources and tobacco smoke. AGEs accumulate in nearly all mammalian tissues and are linked to various health issues, such as diabetes and its related complications, cardiovascular disease, and neurodegenerative disorders. Our data show that *Annurca* apple by-products at different ripening stages differentially counteract AGEs’ formation by inhibiting protein glycation and protect against AGE-induced cytotoxicity in endothelial cells. In particular, the extracts reduce AGE-induced reactive oxygen species (ROS) production, thereby inhibiting MAPK signaling pathways and caspase-3 activation. Moreover, ripening significantly enhances the concentration of bioactive compounds and the extent of cellular protection. This study highlights new beneficial properties of *Annurca* apple extracts and suggests that adopting nutritional interventions may support health and potentially reduce the risk of complications associated with AGE accumulation.

## 1. Introduction

The *Annurca* apple (Malus domestica cv. *Annurca*), a cultivar native to the Campania region and protected by EU PGI designation, is well known for its remarkable nutraceutical profile. Numerous studies have demonstrated that *Annurca* apples possess one of the highest phenolic contents among apple varieties, with a distinctive abundance of procyanidins, chlorogenic acid, flavonols, and dihydrochalcones [1]. These compounds contribute to a strong antioxidant capacity and are associated with multiple biological effects, including modulation of lipid metabolism, improvement of endothelial function, anti-inflammatory activity, and protection against oxidative-stress-mediated cellular damage [2,3,4]. In addition, *Annurca* polyphenols have been implicated in the regulation of glucose homeostasis and in the prevention of metabolic and degenerative disorders, supporting the growing interest in this cultivar as a functional food source [5,6].

The extraction of bioactive compounds from *Annurca* matrices allows for the separation of fractions that differ substantially in phytochemical composition and therefore in biological activity. Polyphenols are not distributed evenly within the fruit: the peel is typically the richest fraction, while the flesh contains lower but still significant amounts [7].

In particular, quantitative analysis, shown in a previous study, demonstrated that polyphenol distribution in *Annurca* apples depends on both tissue and ripening stage. The peel contains the highest levels, especially in ripe fruits, while the flesh, though lower in concentration, remains important due to its predominance in the edible part. Some polyphenols, like epicatechin, catechin, chlorogenic acid, and quercetin hexoside, are present from early stages, whereas others, such as 4-hydroxy-3-benzoic acid and quercetin, appear only during ripening. Overall, polyphenolic composition changes in a tissue-specific manner, reflecting physiological and biochemical processes during fruit development [1].

The valorization of apple peel as a source of high-value nutraceutical compounds also aligns with current sustainability principles. Peel and other apple by-products are a significant portion of agro-industrial waste, despite being particularly enriched in polyphenols with antioxidant, antimicrobial, and anti-inflammatory properties. Their recovery through environmentally sustainable extraction procedures supports a circular-economy model and reduces the environmental impact of fruit processing [8]. In this context, the *Annurca* cultivar, due to its high peel phenolic concentration and the traditional “reddening” process that enhances pigment and flavonoid content, represents an especially promising candidate for waste-based functional ingredient production. In addition to polyphenols, apple peel is also known to contain relevant amounts of non-phenolic bioactive compounds, particularly pentacyclic triterpenoids such as ursolic acid, which accumulate mainly in the cuticular wax layer. Ursolic acid has been reported to exert antioxidant, anti-inflammatory, and vasoprotective activities.

Advanced glycation end-products (AGEs) constitute a naturally occurring, heterogeneous group of compounds that originate from the glycation reaction, a non-enzymatic and non-selective process that begins with the reaction between reducing sugar (or dicarbonyl compounds) and free amino groups on proteins, nucleic acids, and lipids. AGEs are produced as a normal metabolic product, but under certain conditions, their accumulation can lead to the development and progression of chronic diseases. In fact, besides directly altering protein structure and function, AGE accumulation is linked to a sustained inflammatory response and oxidative stress, both of which are implicated in the pathogenesis and progression of several diseases, such as arteriosclerosis, renal failure, neurodegenerative and cardiovascular diseases, cancer, and diabetes with its vascular complications [9,10,11,12]. In particular, AGE formation increases with normal aging and accelerates in states of hyperglycemia and diabetes [13,14]. In diabetes, plasma AGE levels rise markedly due to enhanced glycation driven by hyperglycemia and increased oxidative stress, playing a prominent role in the disease’s pathogenesis and related vascular complications [15,16,17,18,19]. AGE formation amplifies oxidative stress and inflammatory responses and reduces nitric oxide (NO)-mediated vasodilation, contributing to vascular damage in both microvascular (retinopathy, neuropathy, and nephropathy) and macrovascular (ischemic heart disease, cerebrovascular disease, and peripheral vascular disease) contexts [20,21,22,23]. Two main mechanisms are proposed for how AGEs contribute to aging and diabetes-related diseases: (i) binding to specific pro-oxidant and pro-inflammatory receptors on the cell membrane, especially the receptor for advanced glycation end products (RAGE); and (ii) direct alteration of protein structure, properties, and function [9,16]. In particular, AGE–RAGE interaction activates the NADPH oxidase system, increasing intracellular oxidative stress and triggering downstream pathways involved in inflammation, proliferation, and apoptosis, including NF-κB, ERK, p38/MAPK, PI3K-AKT, and JAK/STAT signaling [24].

Considering the primary role of AGEs in health complications, significant efforts have been made to identify anti-glycating and anti-AGE agents, as well as AGE-RAGE axis signaling blockers with the aim of developing new potential supplemental therapeutic strategies to counteract the AGE-induced toxicity. A variety of synthetic chemical AGE inhibitors have been developed to inhibit the formation of AGEs at different steps of glycation reaction [15,25,26]. However, the use of AGE synthetic inhibitors is limited due to safety concerns and their side effects. Recently, plant extract products and/or their isolated bioactive compounds with well-recognized biological properties have attracted increasing interest as anti-AGE agents due to their lower complications compared to synthetic compounds [15,27]. In this respect, plant-derived polyphenolic compounds are known to possess antioxidant and antiglycation activity, as well as anti-inflammatory properties, thus showing great therapeutic potential in minimizing the pathogenesis of secondary complications of diabetes [28,29,30,31]. In particular, much attention has been paid to the identification of compounds able to inhibit ROS signaling pathways in order to counteract the AGE-induced toxicity in endothelial cells with the aim of developing therapeutic strategies in the treatment of diabetic vascular complications [32,33,34]. In this respect, *Annurca* apple extract, for its high phenolic content, possesses a strong antioxidant activity that can protect against oxidative-stress-mediated cellular damage and prevent the associated endothelial disfunction in diabetic vascular complications. In the light of these considerations, in this study, we have analyzed the effect of *Annurca* apple by-products (peel and flesh) at different ripening stages on the AGE-induced apoptosis in human endothelial cell models.

## 2. Materials and Methods

### 2.1. Materials

Human insulin (S-I2643), D-ribose (W379301), (Methylglyoxal (M0252), 3-(4,5-dimethylthiazol-2-yl)-2,5-diphenyl-tetrazolium bromide (MTT) (Sigma-Aldrich Co., St. Louis, MO, USA). Primary antibodies: phospho-p44/42 MAPK (Thr202/Tyr204) #4377), p-44/42 MAPK (3A7) (#9107), Cleaved Caspase-3 (Asp 175) (5A1E) (#9664), β-Tubulin (#2146), and vinculin (#13901) (Cell Signaling Technology, Boston, MA, USA). Eme Oxygenase 1 (#101147) (Gene Tex, Freising, Germany). Secondary antibodies: anti-mouse (#7076) and anti-rabbit (#7074) (Cell Signaling Technology, Boston, MA, USA). All other chemicals were of analytical grade. Methylglyoxal was further purified as previously described [35].

### 2.2. Fruit Collection

*Annurca* apples (*Malus pumila* Mill. cv. *Annurca*) were harvested in 2024 from an orchard located in Giugliano in Campania (Naples, Italy). The fruits were harvested in September in the pre-climacteric phase, characterized by green skin and incomplete ripeness [36]. Some of these unripe apples were at once processed for analytical purposes. The remaining fruits underwent the traditional post-harvest reddening process in “melai”, which consisted of a raised bed of well-drained soil covered with a layer of straw, where the apples were exposed to natural sunlight for about a month. After the reddening phase, samples of ripe fruits were collected and processed for comparative analysis [1].

### 2.3. Polyphenol Extraction and Characterization

Forty grams of *Annurca* apple sample was homogenized by a Tefal rondo 500 homogenizer for 5 min using 40 mL of 80% methanol and 20% acidified water. The aqueous acidic solution was prepared by adding 15 mL of 12 N HCl to 1 L of distilled water. After centrifugation (18,000× *g* for 25 min), the slurry was dried under vacuum by using the Eppendorf Concentrator Plus. The dried extracts were dissolved in 10 mL of PBS and frozen at −80 °C until use. The total polyphenolic content of apple extracts was estimated using the Folin–Ciocalteu phenolic reagent. The extracts (100 μL) were mixed with Folin–Ciocalteu phenolic reagent (0.5 mL), deionized water (0.9 mL), and Na_2_CO_3_ (7.5% *w*/*v*, 4 mL). The absorbance at 765 nm was measured 2 h after incubation at room temperature using a UV-3100PC spectrophotometer. The measurement was compared to a standard curve of prepared catechin solutions and expressed in milligrams of catechin (CAE) equivalent per 100 g FW (fresh weight) of apple sample. Quantification was performed using a standard curve generated from a series of catechin reference solutions (catechin calibration curve).

Polyphenol content was calculated as milligrams of catechin equivalents (CAEq) per 100 g of fresh weight (FW) of apple sample, and as millimolar (mM) concentrations, determined as millimoles of polyphenols (CAEq) per liter of extract. All extract concentrations are reported as catechin equivalents to ensure reliable comparison among samples, regardless of differences in their polyphenolic profiles. It should be noted that the Folin–Ciocalteu assay provides an estimation of total reducing capacity rather than a specific quantification of individual phenolic compounds. So, total phenolic content is expressed as catechin equivalent and does not reflect the true molar concentration of the phenolic mixture. Moreover, non-phenolic reducing substances naturally present in apple matrices, such as ascorbic acid, may contribute to the Folin–Ciocalteu signal, potentially leading to an overestimation of total phenolic content.

### 2.4. AGEs’ Formation

Human insulin was dissolved in phosphate buffer 50 mM to a final concentration of 4 mg/mL at pH 7.0, and protein concentration was determined by absorbance (ε_275_ = 4560 M^−1^cm^−1^). Glycated insulin was prepared by mixing human insulin at a final concentration of 1 mg/mL and 0.5 M D-ribose or 1 mM methylglyoxal (MG), in 50 mM NaH_2_PO_4_ buffer, pH 7.0; passed through a 0.22 μm filter; and incubated at 37 °C in sterile conditions in the absence and in the presence of *Annurca* apple extracts. Human insulin free of glycating agent was used as protein control.

### 2.5. Fluorescence Measurements

Fluorescence measurements were performed on a Perkin Elmer Life Sciences LS 55 spectrofluorometer. To assess the intrinsic fluorescence of AGEs (λ_ex_ 320 nm/λ_em_ 410 nm), glycated insulin at a final concentration of 8 μM was monitored at different incubation times with the glycating agent with and without *Annurca* apple extracts. Tyrosine fluorescence emission (λ_ex_ 275 nm/λ_em_ 305 nm) was measured on 10 µM glycated insulin upon addition of *Annurca* apple extracts at a 1:1 molar ratio.

### 2.6. Cell Cultures and Treatments

EA.HY926 human endothelial cells (CRL-2922, ATCC Virginia, Manassas, VA, USA) were cultured as previously described [3]. The protective effect of different *Annurca* apple fractions was monitored at 24 and 48 h by cell pretreatment with apple extract at different concentrations (0, 25, 50, and 100 µM) and incubation with 30 µM of AGEs (fully glycated insulin). For all experiments, untreated cells and cells incubated in the presence of only *Annurca* apple fractions at the tested concentrations were used as control.

### 2.7. MTT Assay

Cellular metabolic activity was determined through the MTT assay as indicator of cell viability according to a previously described protocol [35]. Data are expressed as percentage reduction of MTT compared with the control ± SD from five different experiments carried out in triplicate.

### 2.8. Trypan Blue Assay

Trypan Blue assay allowed us to estimate the number of dead cells in the experimental groups according to a previously described protocol [35]. Trypan Blue experiments were performed three times in replicates of six wells for each datapoint in each experiment. Data are presented as means ± standard deviation for a representative experiment.

### 2.9. Detection of Intracellular ROS

Measurement of intracellular ROS species was performed using 2′,7′-dichlorofluorescin diacetate (DCFH-DA) assay according to a previously described protocol [28]. DCF fluorescence intensity (λ_ex_ 488 nm/λ_em_ 530 nm) was recorded using Perkin Elmer Life Sciences LS 55 spectrofluorometer. Data are expressed as average ± SD from five different experiments carried out in triplicate.

### 2.10. ABTS Assay

The ABTS assay was employed to determine the total antioxidant capacity (TAC) of the samples. This method is based on the oxidation of ABTS to its radical cation (ABTS^+^), which generates a green color. The presence of antioxidants inhibits this oxidation, resulting in a reduction of absorbance. The assay was conducted according to the manufacturer’s protocol. In brief, 10 μL of cell lysate samples was added to 200 μL of the working solution in the wells of a 96-well microplate. The plate was kept at room temperature for 5 min, and absorbance was subsequently measured at 734 nm using an iMark microplate reader. TAC values were calculated based on a standard curve prepared with Trolox.

### 2.11. Immunoblotting

Protein cellular extracts (25 μg) were separated by SDS-PAGE (10%) under reducing conditions and subsequently transferred onto a polyvinylidene difluoride (PVDF) membrane. The membranes were blocked with 5% non-fat dry milk (A0530; AppliChem) for 1 h at room temperature, followed by overnight incubation at 4 °C with specific primary antibodies. After 1 h of incubation with the appropriate horseradish peroxidase-conjugated secondary antibodies, immunocomplexes were detected using an enhanced chemiluminescence detection kit (Elabscience Biotechnology, Houston, TX, USA) and visualized with the ChemiDoc XR system (Bio-Rad, Hercules, CA, USA). The relative intensity of protein bands was quantified using the Gel Doc XR System (Bio-Rad, Hercules, CA, USA).

### 2.12. Statistical Analysis

Stata software (Version 13.0; StataCorp LP., College Station, TX, USA) was used for statistical analyses. For treatments found significant by analysis of variance (ANOVA), Tukey’s test was performed. Results are presented as mean ± SD, and statistical significance was set at *p* < 0.05.

## 3. Results and Discussion

### 3.1. Annurca Apple Extracts’ Effect on Insulin-AGE Formation

**Figure 1:**
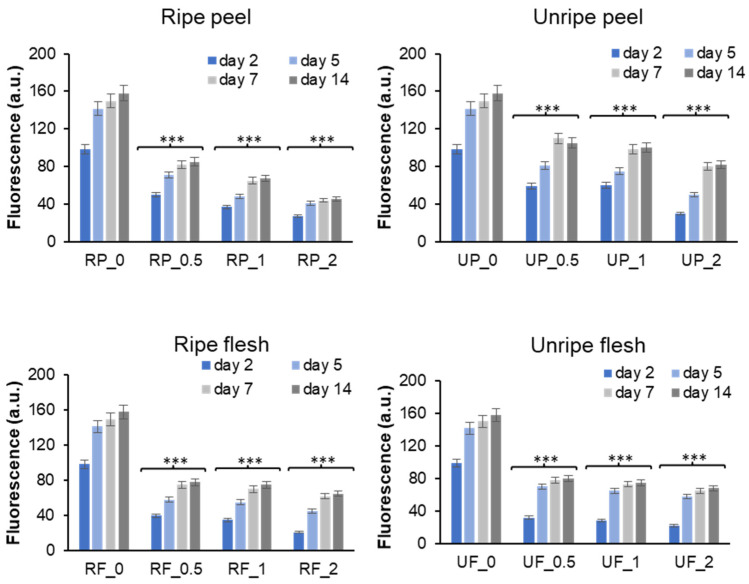
Effect of *Annurca* apple extracts on insulin glycation reaction by D-ribose. Insulin samples were incubated at 37 °C with 0.5 M D-ribose at different concentrations of *Annurca* apple extracts, and AGE fluorescence (λ_ex_ 320 nm/λ_em_ 410 nm) was monitored at different time points. Insulin/apple fraction molar ratio was 1:0 (0), 1:0.5 (0.5), 1:1 (1), and 1:2 (2). RP, ripe peel; UP, unripe peel; RF, ripe flesh; UF, unripe flesh. Other experimental details are described in the Methods section. *** *p* ˂ 0.05 versus sample in the absence of apple fractions.

Insulin is susceptible to glycation by glucose; D-ribose; and other highly reactive carbonyls, such as methylglyoxal (MG), especially in diabetic conditions [37,38,39,40]. Both D-ribose and MG may effectively react with human insulin to produce completely glycated protein in a few days, as we have previously demonstrated [41]. The glycation reaction was carried out both with and without different apple fractions, and AGE production was quantified using fluorescence spectroscopy to investigate the impact of *Annurca* apple extracts on the insulin glycation process. In fact, when AGEs are excited at 320 nm, they typically emit fluorescence at 410 nm [42]. To this aim, insulin samples were incubated at 37 °C with 0.5 M D-ribose (Figure 1) or 1 mM MG (Figure S1) in the presence of different concentrations of apple fractions (at two different ripening stages), and AGE fluorescence was monitored in time. In particular, the four *Annurca* apple fractions tested were ripe and unripe flesh, and ripe and unripe peel, and they were incubated with human insulin at different molar ratios: 0:1, 0.5:1, 1:1, and 2:1.

As expected, insulin glycated by D-ribose without the presence of apple extracts showed a significant emission intensity at 410 nm throughout the incubation period, with the glycation reaction completed in approximately 7 days (Figure 1). In contrast, in the presence of *Annurca* apple fractions, a significant reduction in AGE formation was observed at all incubation times, as indicated by the clear decrease in fluorescence intensity. The inhibition of AGE formation was stronger in the presence of ripe peel, and the 0.5× molar ratio was enough to strongly restrain the process. A different effect was observed for insulin sample glycated by MG. Indeed, in this reaction, the presence of apple fraction did not perturb AGEs’ fluorescence intensity (Figure S1).

**Figure 2:**
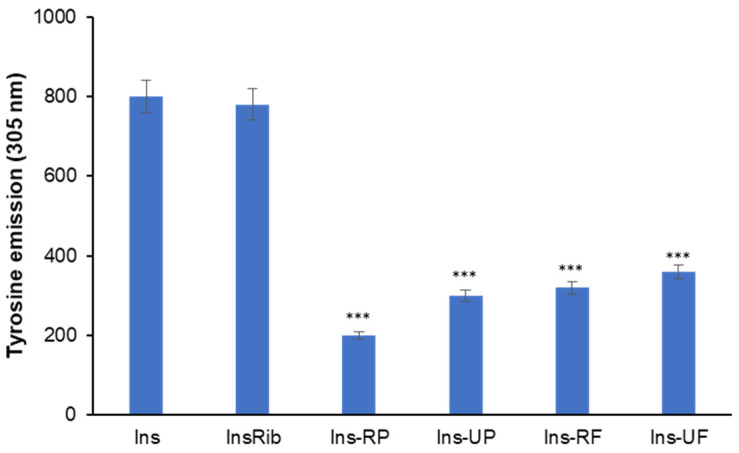
Microenvironment conformational changes in insulin in the presence of apple fractions. Tyrosine fluorescence emission (λ_ex_ 275 nm/λ_em_ 305 nm) was evaluated in native insulin (Ins) and insulin glycated by D-ribose (InsRib) with and without ripe (RP) and unripe peel (UP), ripe (RF), and unripe flesh (UF) fractions at 1:1 molar ratio. Insulin working concentration was 10 µM. Other experimental details can be found in the Methods section. *** *p* ˂ 0.05 versus sample in the absence of apple fractions.

These data show that *Annurca* apple extracts have a differential effect on insulin glycation depending on the glycating agent. Specifically, the extracts significantly inhibited AGE formation in the presence of D-ribose, whereas no inhibitory effect was observed when MG was used. In insulin glycation reaction, D-ribose is known to react with N-terminus and Lys29 of insulin B-chain, while MG with a single site, i.e., Arg22 of insulin B chain [37]. In this respect, the different effect could be ascribed to the distinct insulin glycation sites that could be differently hidden by the apple fraction components. As D-ribose glycation site (LysB29) is very close to Tyr26 in insulin structure, to monitor if apple extracts could reduce the access of D-ribose to this glycation site, we performed the glycation reaction with D-ribose both in the presence and in the absence of ripe peel fraction and recorded the tyrosine emission fluorescence at the end of the glycation process (7 days; Figure 2).

Insulin contains four tyrosyl residues as fluorescence emitters, and its spectrum is characterized by the typical tyrosyl emission centered at 305 nm. Figure 2 shows the emission fluorescence intensity of tyrosines (λ_ex_ 275 nm/λ_em_ 305 nm) in insulin glycated by D-ribose (InsRib) in the presence and in the absence of different apple fractions at a 1:1 molar ratio. In the absence of apple extract, the intrinsic fluorescence of glycated insulin (InsRib) overlaps with that of the native protein (Ins), indicating that glycation does not significantly alter the tyrosyl environment. Differently, the fluorescence signal was strongly decreased in the samples glycated with D-ribose in the presence of apple fractions, thus indicating structural changes leading to quenching of tyrosine emission. Among the four fractions analyzed, the ripe peel shows the strongest effect on tyrosine quenching, and this could be due to an enrichment in this fraction of some molecules (likely polyphenols) able to affect the D-ribose glycation site in insulin structure. This experiment was carried out also in the presence of MG as a glycating agent, but no effect of apple extract on tyrosyl emission was detected.

These data suggest that compounds in the apple extract induce small conformational changes in the tyrosyl environment that could selectively reduce accessibility to the close D-ribose glycation site (LysB29), thus hindering AGEs’ formation. A similar effect has been observed for hydroxytyrosol, a polyphenol that selectively inhibits access to D-ribose insulin glycation site (LysB29) [41].

### 3.2. Annurca Apple Extracts’ Protective Effect in AGE-Induced Toxicity

#### 3.2.1. Evaluation of Cell Toxicity of *Annurca* Apple Extracts at Different Ripening Stages

In order to use *Annurca* apple extracts for cell culture studies, we first studied the cell toxicity of the different fractions at different ripening stages in human endothelial cells. The analysis was performed on different fractions, as the concentration of the individual phenolic compounds within the apple is not steady but depends on distinctive conditions, such as the cultivar, the development of the natural product, the growing conditions, the development, the collection, the storage, and the infections suffered.

**Figure 3:**
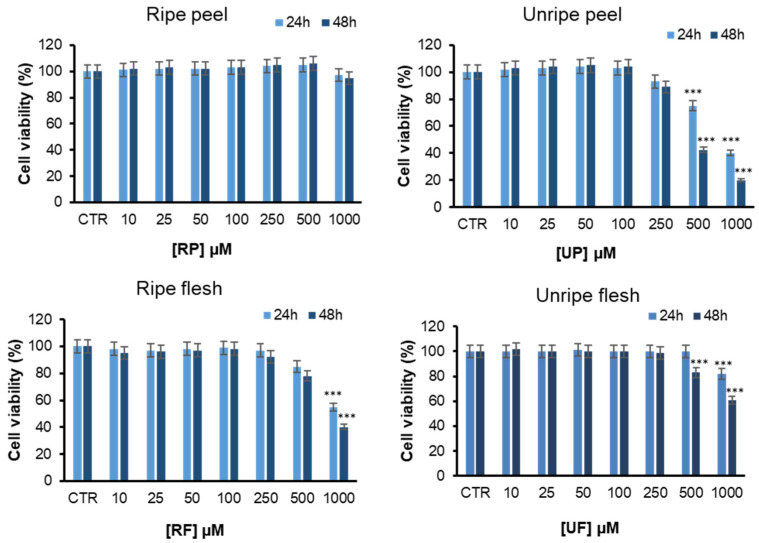
Evaluation of *Annurca* apple extracts’ effect on cytotoxicity in endothelial cells. Cell viability was evaluated by MTT assay in EA.HY926 cells exposed for 24 and 48 h to increasing concentrations (0–1000 µM) of ripe peel (RP), unripe peel (UP), ripe flesh (RF), and unripe flesh (UF) fractions. Data are expressed as average percentage of MTT reduction ± SD relative to untreated cells (CTR). Other experimental details can be found in the Methods section. *** *p* ˂ 0.05 versus CTR.

With the aim of evaluating the cell toxicity for the four different fractions, we performed toxicity studies using EA.hy926 cells, a widely accepted representative endothelial cell line, to improve the experimental conditions. For this aim, EA.hy926 cells were exposed to different concentrations of *Annurca* apple fractions (flesh and peel) at two different ripening stages (ripe and not ripe) for 24 and 48 h, and cell toxicity was evaluated by MTT assay (Figure 3).

Interestingly, no difference in cell viability was observed in cells exposed to different fractions up to 250 µM concentration, indicating that none of these fractions affects cell viability in endothelial cells at micromolar range of concentrations. At higher concentrations (0.5 and 1 mM), while ripe and unripe flesh, as well as unripe peel, were affecting cell viability, ripe peel extract was still not cytotoxic for endothelial cells. The overall data show that the apple extracts possess a good safety profile for endothelial cells, and the ripe peel is non-cytotoxic even at higher concentrations (millimolar range).

#### 3.2.2. *Annurca* Apple Extracts’ Protective Effect in AGE-Induced Toxicity

**Figure 4:**
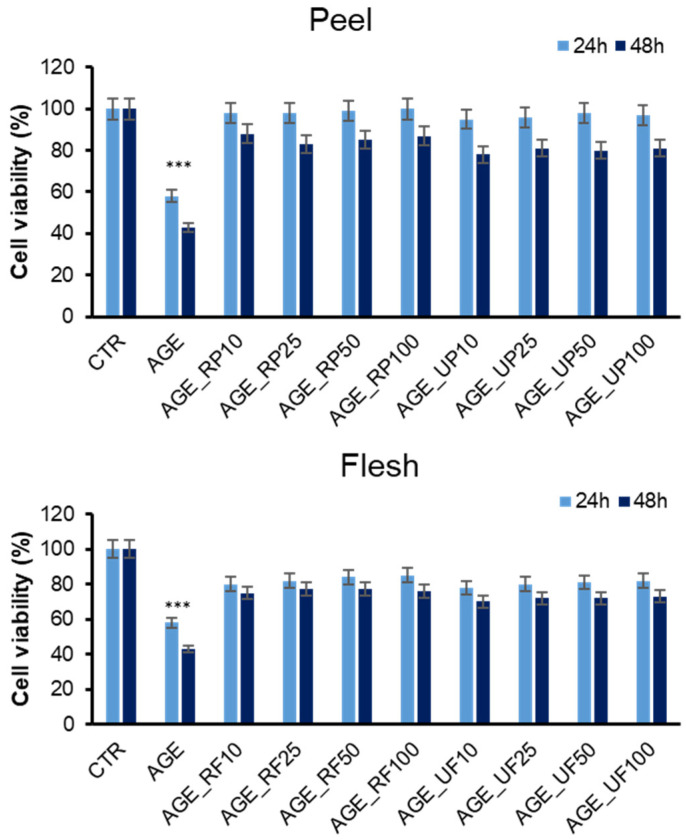
Evaluation of *Annurca* apple extracts’ effect on AGE cytotoxicity in endothelial cells. Cell viability was evaluated by MTT assay in EA.HY926 cells exposed for 24 and 48 h to AGEs and increasing concentrations (0, 10, 25, 50, and 100 µM) of ripe peel (RP), unripe peel (UP), ripe flesh (RF), and unripe flesh (UF) fractions. Data are expressed as average percentage of MTT reduction ± SD relative to untreated cells (CTR) from triplicate wells from 5 separate experiments. Other experimental details are described in the Methods section. *** *p* ˂ 0.05 versus CTR.

*Annurca* apple is rich in phenolic compounds, and it has been associated with a wide range of biological effects, such as free radical scavenging, antimicrobial activity, anti-inflammatory activity, and modulation of oxidative stress-related pathways in vitro [43,44]. Since AGEs are known to induce cytotoxicity by activating inflammatory responses and oxidative stress pathways, the protective effect of *Annurca* apple extracts against AGE-induced toxicity in endothelial cells exposed to fully glycated species was also investigated, as this effect has never been tested before. In particular, the cell viability was tested by the MTT assay in EA.HY926 human endothelial cells pre-incubated with different concentrations of apple fractions and then exposed to AGEs for 24 and 48 h (Figure 4).

**Figure 5:**
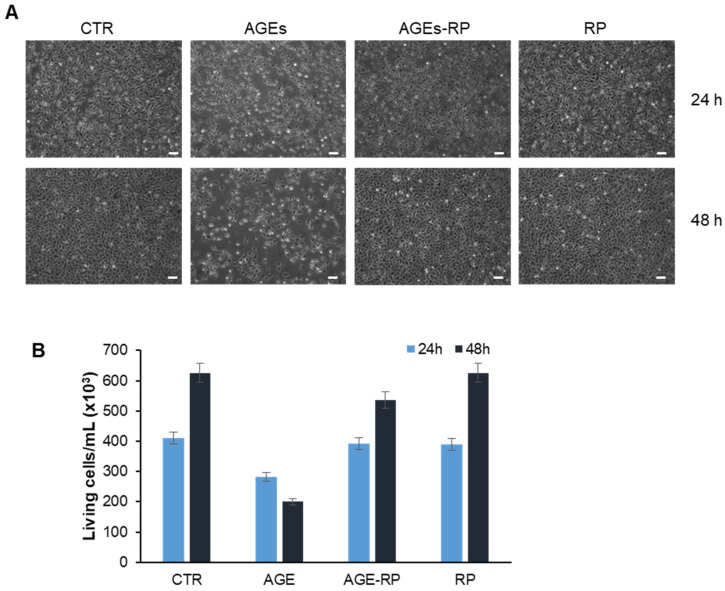
Evaluation of *Annurca* apple extracts’ effect on AGE cytotoxicity in endothelial cells. (**A**) Phase-contrast microscopy images and (**B**) evaluation of living cells for EA.HY926 cells after 24 and 48 h of incubation. CTR, untreated cells; AGE, cells exposed to 30 μM AGE; AGE-RP, cells pretreated with 10 μM ripe peel fraction and then 30 μM AGE; RP, cells treated with 10 μM ripe peel fraction. Scale bar: 200 nm. Data are expressed as average number ± SD relative to untreated cells (CTR) from triplicate wells from 5 separate experiments. Other experimental details are described in the Methods section.

As expected, glycated insulin induced a strong reduction of the cell viability (40% at 24 h, and 60% at 48 h) while, in the presence of *Annurca* apple fractions, no significant reduction was observed at any time point. Interestingly, apple extracts were able to reverse the toxic effect of AGEs even at the lower concentration (10 µM), thus suggesting a strong protective effect on AGE toxicity. The protective effect was stronger for peel compared to flesh fractions, and between ripe and unripe peel, the ripe fraction showed higher protection. To assess changes in cell morphology and cell number following different treatments, the experimental groups were also examined using phase-contrast microscopy (Figure 5). In accordance with the MTT assay, phase-contrast microscopy shows that cells exposed to AGEs for 24 and 48 h display both reductions in cell number and alteration of cell morphology, whereas those pretreated with 10 µM ripe peel fraction exhibit no qualitative and quantitative alterations. Although Figure 5 shows only representative phase-contrast microscopy images obtained using the ripe peel extract, similar effects were observed for all apple extracts; however, the ripe peel fraction still shows a better protective effect (Figure S2). Further confirmation of the protective effect was obtained through the Trypan Blue assay, which allowed us to estimate the number of living and dead cells in all experimental groups (Figure 5B and Figure S2B).

The above data indicate that *Annurca* apple fractions strongly protect endothelial cells by AGE-induced toxicity at low micromolar concentration, and the protective effect is stronger for the ripe peel fraction. As AGE toxicity is mediated by oxidative stress, the different protection observed in the four apple fractions could be associated with their different antioxidant ability. Indeed, the higher protective effect observed for the ripe peel fraction could be directly linked to the higher antioxidant polyphenols’ content in this fraction [3].

#### 3.2.3. *Annurca* Apple Extracts’ Protective Effect in AGE-Induced Oxidative Stress

AGEs are widely recognized to contribute to increased oxidative stress and inflammatory responses through their interaction with the RAGE receptor [33,45,46]. Oxidative stress has been identified as a central mechanism by which AGEs exert cytotoxic effects on endothelial cells, primarily through the elevation of reactive oxygen species (ROS) production and the resulting mitochondrial membrane depolarization, a critical event in the activation of molecular signaling pathways, leading to inflammation and apoptosis [47,48,49].

**Figure 6:**
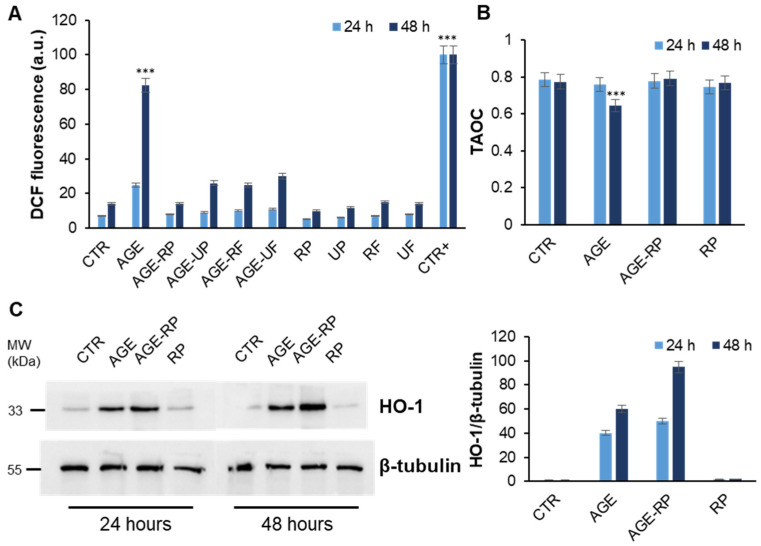
Role of *Annurca* apple extracts AGE-induced oxidative stress. EA.HY926 cells were exposed to 30 µM AGEs and pretreated with 10 µM of ripe peel (RP), unripe peel (UP), ripe flesh (RF), and unripe flesh (UF) fractions, and we evaluated ROS production by DCFHDA assay (**A**) and total antioxidant ability by ABTS assay (**B**) and HO-1 expression (**C**) for samples treated with ripe peel (RP). CTR, untreated cells; AGE, cells treated with 30 µM AGE; CTR+, cells treated with 1.0 mM H_2_O_2_. Data are expressed as average intensity ± SD from triplicate wells from 5 separate experiments. Other experimental details are described in the Methods section. *** *p* ˂ 0.05 versus CTR.

The *Annurca* apple is known for its strong antioxidant activity, primarily due to its high polyphenol content, which efficiently scavenges free radicals and helps reduce oxidative stress in cells, protecting them from ROS-induced damage [50]. In order to identify the molecular basis of the cellular protection by which *Annurca* apple extracts counteract AGE toxicity, we evaluated their effect both on oxidative stress and related cell signaling pathways induced by AGEs. First, we evaluated the effect of different *Annurca* fractions (peel and flesh) at different ripening stages (ripe and unripe) on AGE-associated oxidative damage (Figure 6).

In particular, the ability of *Annurca* fractions to reduce the ROS production associated with AGE treatment was tested. As AGEs are known to induce ROS production in endothelial cells, the intracellular ROS levels were measured in EA.HY926 cells pretreated with 10 μM apple fractions and then treated with AGEs for 24 and 48 h, using DCFHDA fluorescence assay (Figure 6A). Our results show that treatment with AGEs 30 μM promotes an increase in DCF fluorescence after both 24 and 48 h of incubation, indicative of ROS production. By contrast, in the sample pre-incubated for 5 h with different apple fractions, the ROS levels were like those of untreated cells, thus suggesting that *Annurca* extracts are able to counteract the AGE-induced ROS production in EA.HY926 cells. Interestingly, the stronger effect has been observed for cells pretreated with ripe peel extract, and this could be associated with the higher amount of antioxidant polyphenols in this fraction [1]. Further confirmation of the antioxidant activity of the ripe peel was obtained by the ABTS assay in which we evaluated cell antioxidant defense (Figure 6B). At 24 h, no significant differences were observed among control samples, those treated with AGEs, and samples treated with the *Annurca* apple peel, either alone or in combination with AGEs. Differently, after 48 h, a substantial decrease in the antioxidant capacity of cell lysates treated with AGEs was detected, suggesting that prolonged exposure to AGEs impairs the cellular antioxidant defense system. Samples treated with the *Annurca* apple peel in combination with AGEs, however, showed antioxidant levels comparable to controls, indicating that the peel extract effectively counteracts AGE-induced oxidative stress and helps maintain cellular redox homeostasis. Oxidative stress was further assessed by evaluating the expression of heme oxygenase-1 (HO-1), a key mediator in the cellular response to ROS (Figure 6C). Results showed that AGE-treated cells exhibited reduced HO-1 expression, correlating with elevated oxidative stress. Notably, in cells co-treated with AGEs and ripe peel extract, HO-1 levels were comparable to those of untreated controls, indicating that the ripe peel extract effectively mitigates AGE-induced oxidative stress and protects against related cytotoxicity. The antioxidant effect on AGE toxicity seems to be related to the ability to upregulate the HO-1 level in endothelial cells, thus keeping active the cellular antioxidant pathways. In this respect, a plausible mechanistic effect for *Annurca* extract’s protective effect could involve activation of the Nrf2/ARE signaling pathway, which is able to upregulate HO-1 expression, thus promoting cellular antioxidant defense. Nrf2 is a transcription factor that upregulates the expression of several phase II and antioxidant enzymes, such as HO-1 in response to oxidative stress [51]. Many natural polyphenols compounds have been shown to preserve mitochondrial function upon exposure to AGEs and their dicarbonyl precursors. Specifically, the protective effect was partially ascribed to the Nrf2-dependent upregulation of antioxidant enzymes and to the direct scavenging of mitochondrial ROS [35,52,53,54,55].

The effect is more marked for the ripe peel fraction, and this could be associated with the higher concentration of antioxidant polyphenols identified in this fraction [1]. Indeed, *Annurca* apples are characterized by a unique polyphenolic profile, with higher levels of specific flavonoids and phenolic acids, particularly concentrated in the peel, which may explain the stronger antioxidant activity [56].

#### 3.2.4. *Annurca* Apple Extracts’ Protective Effect in AGE-Induced Apoptosis

AGEs also contribute to increased oxidative stress, inflammation, and apoptosis through their interaction with receptor for advanced glycation end products (RAGE). This interaction activates NF-*κ*B, leading to increased expression of pro-inflammatory cytokines and stimulation of the MAPK signaling pathway via phosphorylation of extracellular signal-regulated kinases (ERK1/2) [45,46,57]. Our recent findings demonstrate that glycated insulin triggers NF-*κ*B activation and caspase 3/7 activity in endothelial cells through the AGE-RAGE signaling pathway [58].

**Figure 7:**
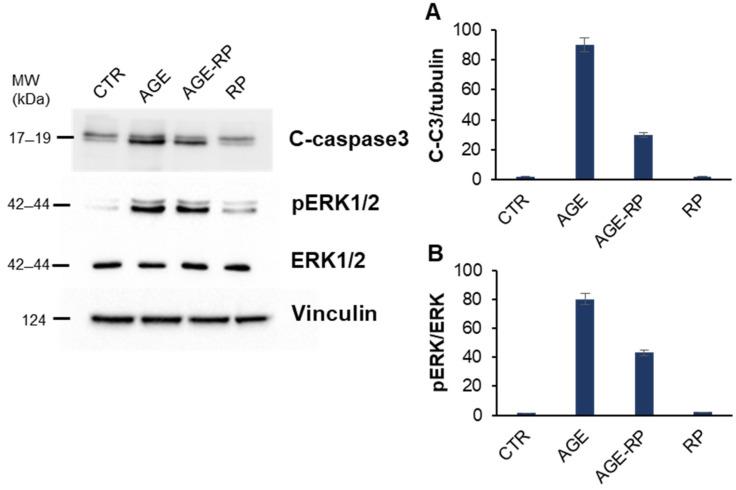
Role of *Annurca* apple extracts in AGE-induced apoptosis. EA.HY926 cells were exposed to AGE (AGE) and pretreated with ripe peel fraction (AGE-RP), and caspase 3 activation (**A**) and pERK phosphorylation (**B**) were evaluated by Western blot analysis. CTR, untreated cells; RP, cells treated with ripe peel fraction. Data are expressed as average ± SD from five independent experiments carried out in triplicate. AGE and RP concentrations were 30 and 10 µM, respectively. Other experimental details can be found in the Methods section.

To further investigate the protective effect of *Annurca* extracts against AGE-induced damage in endothelial cells, Western blot analysis was carried out to assess the potential impact of the extracts on AGE-triggered apoptosis (Figure 7). First, we evaluated the activation of caspase 3 in EA.HY926 cells treated in the presence and in the absence of 10 µM ripe peel extract upon AGE treatment at 48 h by measuring the cleaved active caspase 3 (C-C3). As expected, treatment with AGEs induced caspase 3 cleavage; however, no activation was observed in cells pretreated with the ripe peel fraction, indicating a protective effect against AGE-induced apoptosis in EA.HY926 cells.

To monitor if the protection observed by *Annurca* apple peel on AGE-induced apoptosis occurred through the MAPKs’ signaling pathways, we also evaluated the activation of ERK1/2 MAPKs in the same experimental groups (Figure 7). As expected, the Western blot analysis suggests that MG promotes activation of ERK1/2 at 48 h incubation in EA.HY926 cells, and this is consistent with the observed caspase-3 activation. Differently, in cells pre-incubated with *Annurca* ripe peel fraction, no activation of ERK1/2 is observed, thus suggesting that apple extract is able to protect EA.HY926 cells by interfering with MAPKs pro-apoptotic pathways. Different apple polyphenols have been shown to exert anti-inflammatory effects through the inhibition of the RAGE/p38 MAPK/NF-κB signaling pathway [59]. In particular, phloretin, an apple polyphenol, is able to protect via AGE-induced inflammation through the RAGE/p38 MAPK pathway [60]. Moreover, data obtained with flavonoid-rich apple extract showed inhibition of oxidant-related pathways in endothelial cells mediated by NF-*κ*B signaling [61].

These data show that, while AGEs triggers cell apoptosis in endothelial cells by promoting phosphorylation of ERK1/2 and caspase-3 activation, pretreatment with ripe peel fraction promotes a significant decrease in the MAPKs’ phosphorylation and suppresses apoptosis. The process of ROS-induced cellular production is as a key link between oxidative stress and apoptosis. Elevated levels of AGEs promote apoptosis in endothelial cells by triggering mitochondrial dysfunction and activating ROS/MAPK/Nf-kB signaling pathways, as well as inducing endoplasmic reticulum (ER) stress [34,62,63]. Our data suggest that the beneficial effect of *Annurca* extracts in the AGE-related toxicity is mainly associated with its antioxidant activity. The ability of *Annurca* apple fractions to maintain redox balance appears to be the crucial mechanism for preventing apoptosis in endothelial cells.

## 4. Conclusions

This study demonstrates that both the peel and flesh of *Annurca* apple effectively inhibit AGEs’ formation and protect endothelial cells from AGE-induced cytotoxicity by reducing intracellular ROS production. This protection against oxidative stress subsequently prevents cellular apoptosis through the suppression of the ERK 1/2 MAPKs’ signaling pathways. The ability of *Annurca* apple fractions to maintain redox balance appears to be the crucial mechanism for preventing apoptosis in endothelial cells. In this context, our study highlights a promising protective effect of *Annurca* apple against dicarbonyl-induced endothelial toxicity. Among the various fractions analyzed, the ripe peel emerged as the most effective in counteracting AGE-induced toxicity. The differential biological efficacy is likely related to qualitative differences in the polyphenolic phytocomplex, rather than a mere dose-dependent effect. Indeed, polyphenols are not uniformly distributed across different apple tissues, and the peel is characterized not only by a higher content but also by a qualitatively distinct profile. Moreover, the ripening process does not simply increase the overall quantity of phenolic compounds but also induces modifications in their composition, favoring the accumulation of molecules with enhanced antioxidant activity.

In addition, the phenolic content and composition are strongly influenced by cultivation conditions, including soil type, climate, irrigation, and agronomic practices, as well as by post-harvest handling and ripening stage. These factors can modulate both the quantity and quality of bioactive compounds, potentially affecting their efficacy in counteracting AGEs’ formation and oxidative stress. Therefore, the pronounced protective effect of *Annurca* apple extracts, especially from the ripe peel, should be interpreted within this cultivar- and environment-specific context, highlighting the importance of both genetic and agronomic factors in determining the functional potential of apple-derived phytocomplexes. These findings reveal new beneficial properties of *Annurca* apple extract and suggest that implementing nutritional interventions can help maintain overall health and reduce the risk of AGE-related complications.

From a sustainability perspective, the identification of the ripe peel as the biologically most active fraction has strategic value. Indeed, the peel represents a widely available by-product that is often discarded during agro-industrial processing. Its valorization as a source of high-value functional extracts aligns fully with the principles of the circular economy, enabling both waste reduction and the development of natural ingredients that are potentially useful in the prevention of AGE-related pathologies.

## Data Availability

The data presented in this study are available upon request from the corresponding author.

## Supplementary Materials

The following supporting information can be downloaded at https://www.mdpi.com/article/10.3390/antiox15020200/s1, Figure S1: Effect of apple extracts on insulin glycation reaction by MG; Figure S2: Evaluation of *Annurca* apple extracts on AGE cytotoxicity in endothelial cells.
