# Supplementary material for: Annurca Apple By-Products at Different Ripening Stages Inhibit AGE Formation and Protect Against AGE-Induced Cytotoxicity Through Antioxidant Activity
Source: Antioxidants (Basel). 2026 Feb 3;15(2):200. doi: 10.3390/antiox15020200 (PMC12937829; doi:10.3390/antiox15020200)
Supplement: Supplementary file 1 [file antioxidants-15-00200-s001.zip › antioxidants-4109792-supplementary.pdf]

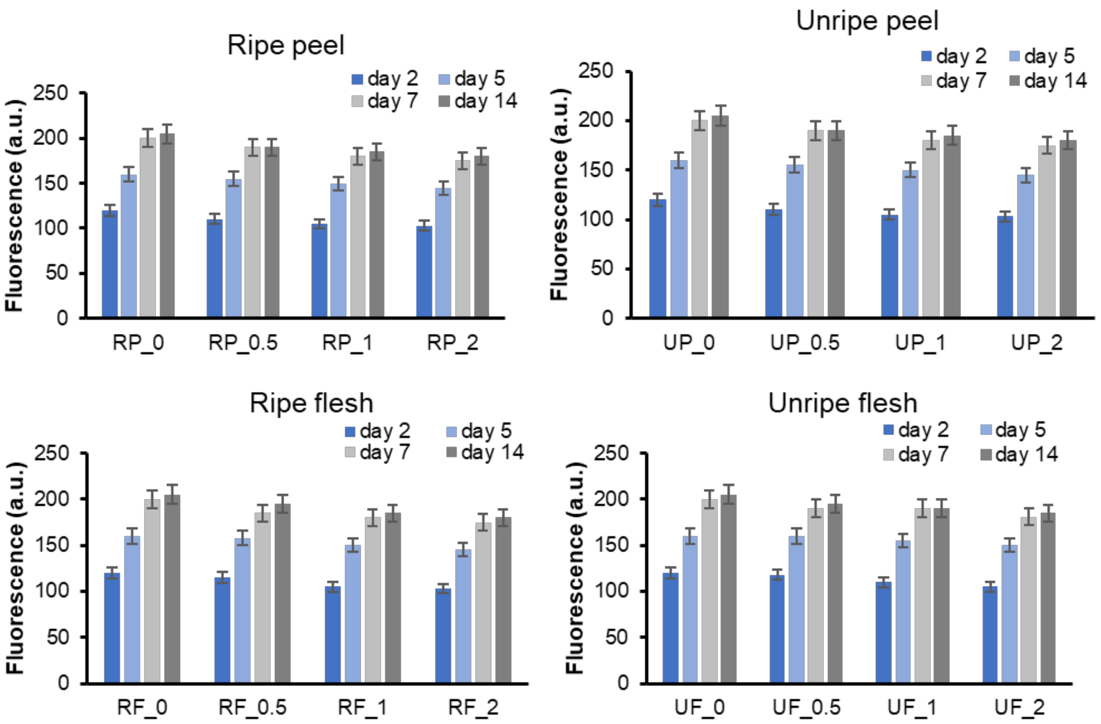

Figure S1. Effect of apple extracts on insulin glycation reaction by MG. Insulin samples were incubated at 37 °C with 5 mM MG at different concentrations of Annurca apple extracts and AGE fluorescence (λ_{ex} 320 nm/λ_{em} 410 nm) was monitored at different time points. Insulin/apple fraction molar ratio was 1:0 (0), 1:0.5 (0.5), 1:1 (1), and 1:2 (2). RP, ripe peel; UP, unripe peel; RF, ripe flesh; UF, unripe flesh. Other experimental details are described in the Methods section.

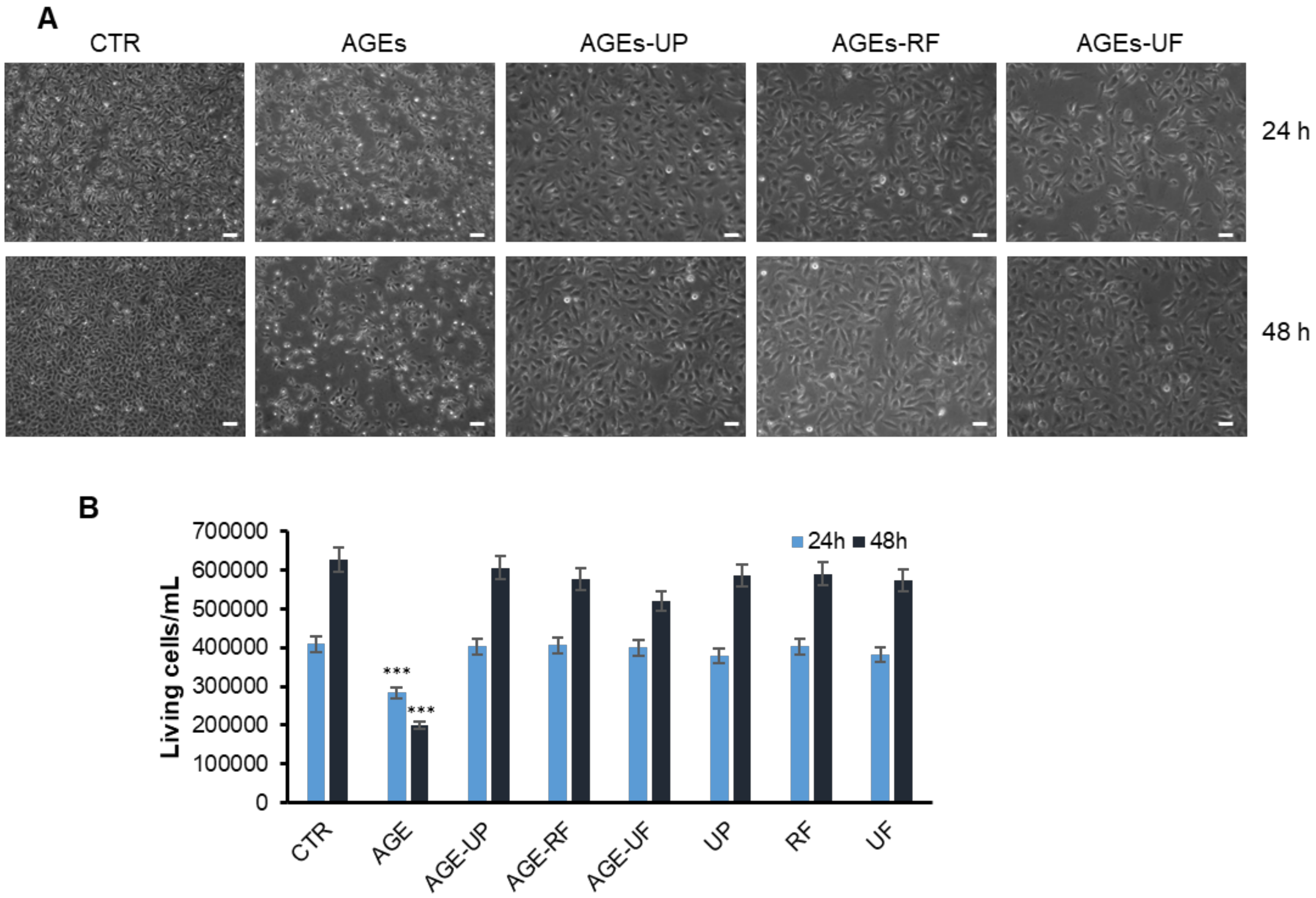

Figure S2. Evaluation of *Annurca* apple extracts on AGE cytotoxicity in endothelial cells. (A) Phase-contrast microscopy images and (B) evaluation of living cells for EA.HY926 cells after 24 and 48 hours of incubation. CTR, untreated cells; AGE, cells exposed to 30 μM AGE; AGE-UP, cells pretreated with 10 μM unripe peel fraction and then AGE 30 μM; AGE-RF, cells pretreated with 10 μM ripe flesh fraction and then AGE 30 μM; AGE-UF, cells pretreated with 10 μM unripe flesh fraction and then AGE 30 μM; UP, RF, and UF, cells treated with 10 μM unripe peel, ripe flash, and unripe flash fraction, respectively. Scale bar: 200 nm. Data are expressed as average number ± SD relative to untreated cells (CTR) from triplicate wells from 5 separate experiments. Other experimental details are described in the Methods section. ***$p<0.001$ versus CTR.
